# Anticholinergic Drug Exposure Increases the Risk of Delirium in Older Patients Undergoing Elective Surgery

**DOI:** 10.3389/fmed.2022.871229

**Published:** 2022-05-06

**Authors:** Matthias L. Herrmann, Cindy Boden, Christoph Maurer, Felix Kentischer, Eva Mennig, Sören Wagner, Lars O. Conzelmann, Bernd R. Förstner, Michael A. Rapp, Christine A. F. von Arnim, Michael Denkinger, Gerhard W. Eschweiler, Christine Thomas

**Affiliations:** ^1^Department of Neurology and Neurophysiology, Medical Center-University of Freiburg, Freiburg, Germany; ^2^Geriatric Center and Department of Psychiatry and Psychotherapy, Tübingen University Hospital, Tübingen, Germany; ^3^Center for Geriatrics and Gerontology, University Medical Center Freiburg, Freiburg, Germany; ^4^Department of Geriatric Psychiatry and Psychotherapy, Klinikum Stuttgart, Stuttgart, Germany; ^5^Department of Anesthesiology, Klinikum Stuttgart, Stuttgart, Germany; ^6^Department of Anesthesia, Critical Care and Pain Medicine, Beth Israel Deaconess Medical Center, Harvard Medical School, Boston, MA, United States; ^7^Helios Clinic for Cardiac Surgery, Karlsruhe, Karlsruhe, Germany; ^8^Department of Social and Preventive Medicine, University of Potsdam, Potsdam, Germany; ^9^Department of Geriatrics, University Medical Center Göttingen, Georg August University, Göttingen, Germany; ^10^Geriatric Center, Agaplesion Bethesda Clinic Ulm, Ulm, Germany; ^11^Institute for Geriatric Research, Ulm University Medical Center, Ulm, Germany

**Keywords:** delirium, acute encephalopathy, surgery, anticholinergic, geriatric, postoperative

## Abstract

**Introduction:**

Postoperative delirium (POD) is a common and serious adverse event of surgery in older people. Because of its great impact on patients' safety and quality of life, identification of modifiable risk factors could be useful. Although preoperative medication intake is assumed to be an important modifiable risk factor, the impact of anticholinergic drugs on the occurrence of POD seems underestimated in elective surgery. The aim of this study was to investigate the association between preoperative anticholinergic burden and POD. We hypothesized that a high preoperative anticholinergic burden is an independent, potentially modifiable predisposing and precipitating factor of POD in older people.

**Methods:**

Between November 2017 and April 2019, 1,470 patients of 70 years and older undergoing elective orthopedic, general, cardiac, or vascular surgery were recruited in the randomized, prospective, multicenter PAWEL trial. Anticholinergic burden of a sub-cohort of 899 patients, who did not receive a multimodal intervention for preventing POD, was assessed by two different tools at hospital admission: The established Anticholinergic Risk Scale (ARS) and the recently developed Anticholinergic Burden Score (ABS). POD was detected by confusion assessment method (CAM) and a validated post discharge medical record review. Logistic regression analyses were performed to evaluate the association between anticholinergic burden and POD.

**Results:**

POD was observed in 210 of 899 patients (23.4%). Both ARS and ABS were independently associated with POD. The association persisted after adjustment for relevant confounding factors such as age, sex, comorbidities, preoperative cognitive and physical status, number of prescribed drugs, surgery time, type of surgery and anesthesia, usage of heart-lung-machine, and treatment in intensive care unit. If a patient was taking one of the 56 drugs listed in the ABS, risk for POD was 2.7-fold higher (OR = 2.74, 95% CI = 1.55–4.94) and 1.5-fold higher per additional point on the ARS (OR = 1.54, 95% CI = 1.15–2.02).

**Conclusion:**

Preoperative anticholinergic drug exposure measured by ARS or ABS was independently associated with POD in older patients undergoing elective surgery. Therefore, identification, discontinuation or substitution of anticholinergic medication prior to surgery may be a promising approach to reduce the risk of POD in older patients.

## Introduction

Delirium is a neuropsychiatric syndrome defined by acute decline and fluctuation of attention, cognitive function, and disturbance of awareness (1). Especially in older patients, delirium is a common and serious adverse event of surgery (2) with an incidence ranging from 11 to 51% (3, 4). Postoperative delirium (POD) in older people is often associated with persistent cognitive dysfunction, dementia, higher rates of institutionalization, and increased morbidity and mortality (5, 6). Its etiology is believed to be multifactorial. Besides neuro-inflammation and blood-brain barrier leakage, a neurotransmitter disbalance in terms of acetylcholine deficiency and dopamine excess is thought to be involved in the pathogenesis of delirium (7). Electroencephalographic changes in delirium such as occipital slowing are also indicative of acetylcholine deficiency (8). Multiple pre- and perioperative risk factors are known to predispose to delirium. Preoperative factors comprise age, multimorbidity, frailty, polypharmacy, and deficits in cognitive, sensory, and mobility function (9, 10). Perioperative parameters that have been identified are, for example, type of surgery and anesthesia, surgery time and treatment in intensive care units (2, 9). Because delirium negatively affects patients' safety and quality of life, identifying modifiable risk factors could be of great relevance for its prevention, especially in older patients (11–13). In this regard, preoperative medication use is considered one of the most important potentially modifiable factors in the prevention of POD (14).

Assuming that delirium might be precipitated by an imbalance in cerebral neurotransmission specifically including acetylcholine deficiency (15), drugs with anticholinergic properties (DAPs) could have a significant impact on POD. DAPs are frequently prescribed in older people for a variety of indications such as minor and major depression, bladder disorders, or nausea (16). The cumulative effect of all DAPs taken regularly by an individual is often referred to as the anticholinergic burden (17). However, numerous studies indicated adverse effects of DAPs on cognitive and physical function (18, 19). Anticholinergic burden has been associated with delirium in several settings (18, 20, 21), although results were conflicting (22). Studies on the effect of DAPs on POD are less frequent, and their results were also inconsistent, with one positive (23) and two negatives studies (24, 25). Different results could be caused by the use of different scores calculating anticholinergic burden, small sample sizes, and different definitions of delirium. Therefore, in this study, we used the established Anticholinergic Risk Scale (ARS), which was most consistently associated with delirium (22). In addition, we applied a new, promising score developed in a very large sample of 250,000 participants to assess associations between long-term anticholinergic medication use and the risk of dementia (16). We hypothesized that a high preoperative anticholinergic burden is an independent, potentially modifiable predisposing and precipitating factor for POD in older people.

## Methods

### Study Design

This study is based on a secondary analysis of data collected from 1,470 patients between November 2017 and April 2019 for the PAWEL-Study (Patient safety, cost-effectiveness and quality of life: reduction of delirium risk and postoperative cognitive dysfunction after elective procedures in older adults). The complete protocol for this randomized, prospective, multicenter study has been described in detail previously (12). Briefly, inclusion criteria comprised patients aged 70 years and older scheduled for elective surgery with an expected duration of surgery of at least 60 min. Surgical procedures included orthopedic, general, cardiac, and vascular surgery conducted at five medical centers in the southwest of Germany. Exclusion criteria were life expectancy <15 months, insufficient knowledge of German language, and recently diagnosed severe dementia without a legal representative. We included the sub-cohort of 899 participants of the baseline group who did not receive a multimodal intervention for preventing POD analogous to the PAWEL risk factor study (10).

### Data Collection in the Pre-, Peri-, and Postoperative Phase

Demographic and clinical data were collected at baseline, no more than 3 weeks before the scheduled surgery, including medical history by Charlson Comorbidity Index (26), cognitive screening by Montreal Cognitive Assessment [MoCA, (27)], preoperative physical status by classification of the American Society of Anesthesiologists [ASA, (28)], and functional status by Barthel Index (29) as well as nutritional condition by Body Mass Index. Preoperative depression and anxiety symptoms were assessed by the Patient Health Questionnaire (30). Perioperative data included premedication, surgical procedure with or without cardiopulmonary bypass, cut-to-suture time, and type of anesthesia. Parameters were collected from anesthesia and surgery protocols.

### Anticholinergic Drug Exposure

Each patient's medication was analyzed on admission using the medication list and personal medical history. Long-term medications as well as “as-needed” (“PRN”) medication were included in the analysis if they were taken more than 3 days a week. In view of the high proportion of “over-the-counter”-drugs, the use of sleeping pills was explicitly queried. Preoperative anticholinergic burden was calculated for each patient using the Anticholinergic Risk Scale [ARS (31)]. Briefly summarized, the ARS is a weighted score developed by Rudolph et al. (31), which often has been used in studies investigating the association between anticholinergic burden and delirium in different study populations (21). It comprises 49 DAPs evaluated from 0 (no or low anticholinergic activity) to 3 (highest anticholinergic activity). The sum of all values provides the patient's individual anticholinergic burden. However, the ARS has not been updated since 2008 and the weights of different DAPs are a matter of debate (22). We therefore added the anticholinergic score recently published by Coupland et al. (16). This measure is primarily based on the score of Gray et al. (32) which comprises medications with strong anticholinergic properties identified by the American Geriatrics Society Beers Criteria Update Expert panel (33). Originally, this anticholinergic score was intended to assess the cumulative anticholinergic burden to evaluate associations between long-term anticholinergic drug exposure and the risk of dementia in a large cohort (16). Hereinafter, this score is called Anticholinergic Burden Score (ABS). The ABS includes 56 DAPs of different subgroups which previously have been described in detail (16). In contrast to the ARS, the ABS does not weigh anticholinergic properties of medications but counts the number of received DAPs. Therefore, in this study, we define the preoperative anticholinergic burden as the sum of all DAPs according to the ABS. We did not include the cumulative dosage of the identified DAPs. To our best knowledge, the ABS is used to investigate the association between anticholinergic burden and POD here for the first time.

### Outcome Measures

Primary outcome measure was the occurrence of POD after elective surgery. POD was evaluated daily for up to 7 postoperative days by independent and previously trained assessors (10, 12) using the Confusion Assessment Method in a German operationalized version [CAM, (34, 35)]. Additionally, a chart review based on the DSM-V criteria for delirium (1) was conducted by experienced physicians at discharge. Similar to the SAGES Study (36), POD was defined if at least one of the methods indicated delirium (37).

### Statistical Analysis

Differences between patients with and without POD were evaluated for categorical variables by χ^2^ test and for non-normally distributed continuous variables by Mann-Whitney *U* test. Logistic regression analysis was used to investigate the independent association of preoperative anticholinergic burden and the incidence of POD (Odds Ratios with 95% confidence intervals). First, univariate logistic regression was performed for both, ARS and ABS. In a second step, the model was adjusted for items that were found to be strong confounders in previous studies (2, 4, 10). These were age, sex, Charlson Comorbidity Index, preoperative cognitive status (MoCA), physical status (ASA), number of prescribed drugs, preoperative serum creatinine, surgery time, type of surgery and anesthesia, usage of heart-lung-machine, and treatment in intensive care unit. Missing data in confounding variables led to the exclusion of 53 patients from the adjusted multivariate analysis, which was finally performed on 846 patients. Data were analyzed with the software IBM SPSS Statistics Version 25 (IBM Corporation, Armonk, NY, USA). Results were considered statistically significant at a level of *p* < 0.05.

## Results

### Participants' Characteristics

The sociodemographic and clinical characteristics of all 899 patients aged 70 and older are described in Table 1. The mean age of the participants was 77.3 years (range 70–98 years), and 49.4% were female. According to the Charlson Comorbidity Index, 282 patients (31.4%) had at least three diseases. Participants were taking a median of six drugs with 56.6% taking ≥5 drugs. Most of the enrolled patients had orthopedic surgery (e.g., hip, knee, and spine, *n* = 474, 52.7%). Another group of 377 patients (37.5%) received cardiovascular interventions with or without cardiopulmonary bypass. The remaining 88 patients (9.8%) underwent general surgery (e.g., abdominal surgery). Surgeries were mostly performed under general anesthesia (*n* = 726, 80.8%).

**Table 1:** Sociodemographic and clinical characteristics of enrolled patients.

	**Overall sample**	**No POD**	**POD**	***p***
		***n*** **= 689 (76.6%)**	***n*** **= 210 (23.4%)**	
Age, in years, mean (SD)
*n = 899*	77.3 (4.9)	77.2 (4.8)	77.8 (5.2)	0.153
Female sex, *n* (%)
*n = 899*	444 (49.4)	353 (51.2)	91 (43.3)	**0.045**
Education in years, mean (SD)				
*n = 882*	12.2 (3.0)	12.3 (3.0)	12.2 (2.9)	0.375
MoCA, mean (SD)				
*n = 874*	23.4 (3.9)	23.9 (3.4)	21.7 (4.8)	**<0.001**
Charlson Comorbidity Index, median (IQR)				
*n = 899*	2.0 (0.0–3.0)	2.0 (0.0–3.0)	2.0 (1.0 - 3.0)	**0.004**
Barthel index, *n* (%)				
= 100	596 (68.0)	464 (68.6)	132 (65.7)	0.368
= 85–95	183 (20.9)	142 (21.0)	41 (20.4)	
<85	98 (11.1)	70 (10.4)	28 (13.9)	
*n = 877*				
Number of preoperative medications, median (IQR)
*n = 899*	6.0 (4.0–9.0)	6.0 (4.0–8.0)	7.0 (5.0–9.0)	**<0.001**
PHQ-4, median (IQR)
*n = 870*	1.0 (0–3)	1.0 (0–6)	1.0 (0–6)	0.568
Preoperative benzodiazepines, *n* (%)
*n = 894*	237 (26.4)	174 (25.4)	63 (30.1)	0.174
Preoperative creatinine in mg/l, median (IQR)
*n = 881*	0.9 (0.8–1.1)	0.9 (0.7–1.1)	0.9 (0.8–1.2)	**0.014**
BMI in kg/m^2^, median (IQR)
*n = 885*	26.64 (24.09–29.81)	26.66 (24.03–29.93)	26.64 (24.22–29.73)	0.861
ASA classification, *n* (%)
ASA 1 + 2	246 (27.7)	227 (33.4)	19 (9.2)	**<0.001**
ASA 3 + 4	641 (72.3)	453 (66.6)	188 (90.8)	
*n = 887*				
Surgery type, % orthopedic
Cardiovascular	474 (52.7)	400 (58.1)	74 (35.2)	**<0.001**
Visceral/general	337 (37.5)	216 (31.3)	121 (57.6)	
*n = 899*	88 (9.8)	73 (10.6)	15 (7.2)	
Surgery time in min., mean (SD)				
*n = 898*	146.6 (84.7)	133.1 (74.5)	191.1 (100.0)	**<0.001**
General anesthesia, *n* (%)
*n = 892*	726 (80.8)	545 (79.1)	181 (86.2)	**0.023**
Stay at ICU/IMC, *n* (%)
*n = 893*	538 (60.2)	368 (53.8)	170 (81.3)	**<0.001**
Cardiopulmonary bypass, *n* (%)
*n = 894*	243 (27.2)	147 (21.5)	96 (45.9)	**<0.001**

*SD, standard deviation; IQR, interquartile range; MoCA, Montreal Cognitive Assessment; ICU, Intensive Care Unit; IMC, Intermediate Care Unit; BMI, Body Mass Index; ASA, American Society of Anesthesiologists; PHQ-4, Patient Health Questionnaire. Significant p-values are marked in bold*.

POD was observed in 210 (23.4%) patients. Patients diagnosed with POD took significantly more medications (*p* < 0.001), had more comorbidities (*p* = 0.004), and revealed a lower MoCA level at baseline assessment (*p* < 0.001). Furthermore, patients with POD had a longer surgery time (*p* < 0.001), and were admitted to an intensive care unit more often (*p* < 0.001). POD occurred significantly more often in patients requiring cardiopulmonary bypass (*p* < 0.001). General anesthesia was performed more frequently in patients with POD (*p* = 0.023). POD and non-POD groups did not differ statistically in Body Mass Index, educational level, Barthel Index, preoperative depression, anxiety or treatment with benzodiazepines (see Table 1).

### Association Between Preoperative Anticholinergic Burden and POD

**Figure 1:**
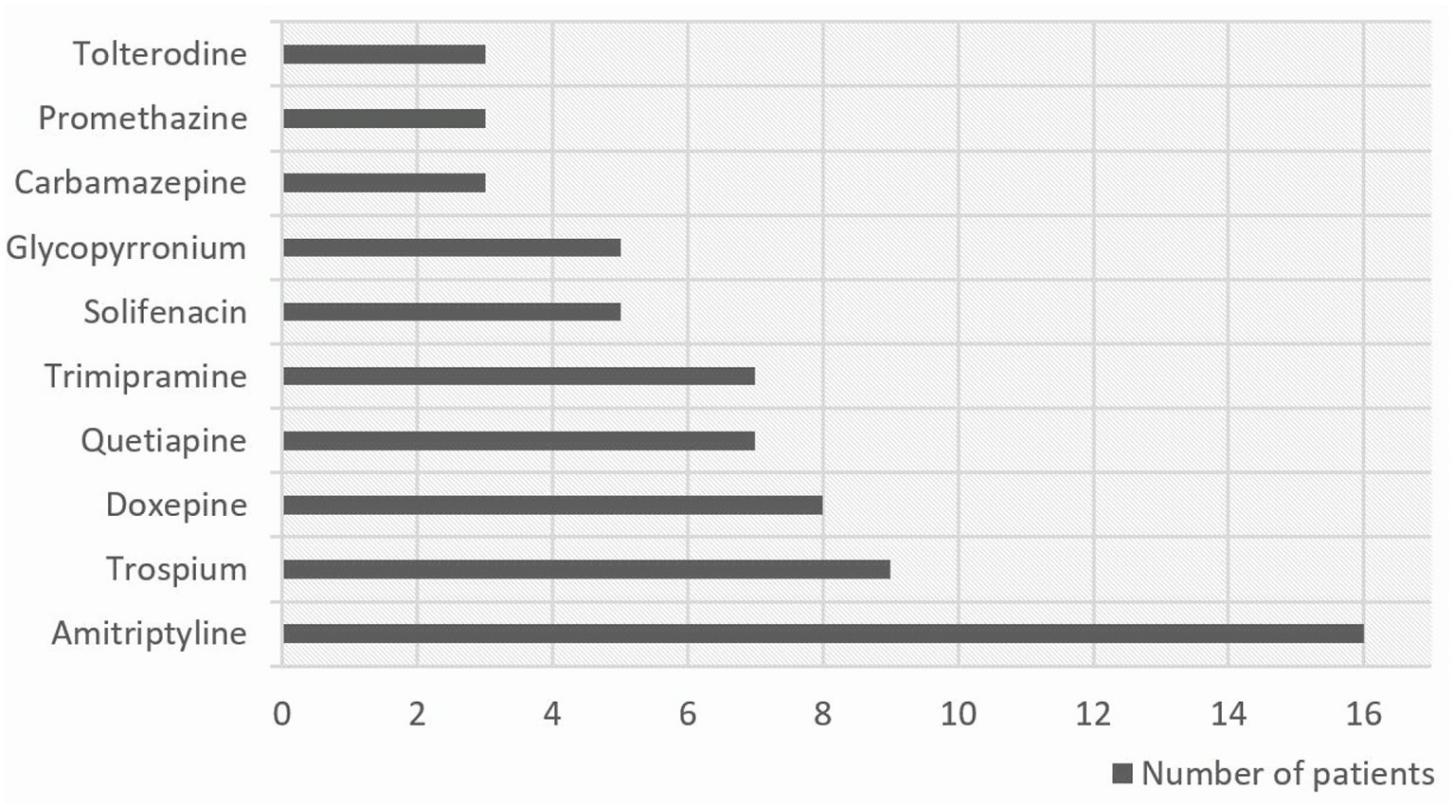
The ten most prescribed DAPs in our study population according to the Anticholinergic Burden Score (ABS).

Using the ARS score, 81 patients (9%) were taking at least one anticholinergic drug. Patients with POD had a significantly higher ARS value than patients without POD (*p* = 0.004). Evaluating medication according to ABS criteria, 71 patients (8%) were taking DAPs. Comparing the POD and non-POD groups, the rate of DAPs was significantly higher in patients with POD (ABS = 1: 6 vs. 11%, ABS = 2: 0 vs. 7%). The most frequently prescribed DAP in both groups (according the ABS criteria) was amitriptyline (*n* = 8 in each group) followed in the delirium group by doxepine (*n* = 4) and solifenacin (*n* = 3). In patients without POD, trospium (*n* = 7) and trimipramine (*n* = 6) were the most commonly used DAPs, in addition to amitriptyline. The top 10 DAPs prescribed are shown in Figure 1.

Although the number of patients taking DAPs and the number of different DAPs (ARS: *n* = 22, ABS: *n* = 19) was relatively small in our study population, we found significant group differences. This was the case when ARS was used and when ABS criteria were applied. In the subgroup of 71 patients who received one or two ABS drugs, the POD rate was 42.3% (*n* = 30/71) and thus almost twice as high as in the group without ABS medication where POD occurred in only 21.7% (*n* = 180/828, see Table 2).

**Table 2:** Characteristics of anticholinergic burden according to ARS and ABS in patients with and without postoperative delirium.

	**Overall sample**	**No POD**	**POD**	***p***
	***n*** **= 899**	***n*** **= 689 (76.6%)**	***n*** **= 210 (23.4%)**	
ARS, mean ± SD (range)	0.17 ± 0.63 (0–5)	0.13 ± 0.50 (0–3)	0.32 ± 0.93 (0–5)	**0.004**
No AB (ARS = 0), *n* (%)	818 (91.0)	637 (92.5)	181 (86.2)	**<0.001**
Moderate AB (ARS = 1), *n* (%)	38 (4.2)	26 (3.8)	12 (5.7)	
Strong AB (ARS = 2), *n* (%)	19 (2.1)	16 (2.3)	3 (1.4)	
High AB (ARS ≥ 3), *n* (%)	24 (2.7)	10 (1.4)	14 (6.7)	
ABS = 0	828 (92.1)	648 (94)	180 (85.7)	**<0.001**
ABS = 1	64 (7.1)	41 (6.0)	23 (11.0)	
ABS = 2	7 (0.8)	0 (0,0)	7 (3.3)	

*SD, standard deviation; IQR, interquartile range; ARS, Anticholinergic Risk Scale; AB, anticholinergic burden; ABS, Anticholinergic Burden Score. Significant p-values are marked in bold*.

Univariate logistic regression analysis revealed a positive association with the occurrence of POD for both scales, ARS and ABS. Odds ratios (ORs) and corresponding 95% confidence intervals (CI) are provided in Table 3. After adjustment for confounding variables, ORs changed only marginally and the positive association with the occurrence of POD persisted. Each additional point in the ARS was associated with a 1.5-fold higher risk of developing POD (OR = 1.54, 95% CI = 1.15–2.02). Applying the ABS criteria resulted in a 2.7 higher likelihood of POD (OR = 2.74, 95% CI = 1.55–4.94) for each additional DAP.

**Table 3:** Odds ratios for postoperative delirium according to ARS and ABS.

	**Model 1: Univariate model**		**Model 2: Adjusted model**	
**Variable**	**OR (95% CI)**	***p***	**OR (95% CI)**	***p***
ARS	1.49 (1.21–1.85)	**<0.001**	1.54 (1.15–2.02)	**0.002**
ABS	2.76 (1.77–4.30)	**<0.001**	2.74 (1.55–4.94)	**0.001**

*OR, odds ratio; using no anticholinergic burden as reference. CI, confidence interval; ARS, Anticholinergic Risk Scale; ABS, Anticholinergic Burden Score*.

*Model 2 was adjusted for age, sex, preoperative cognitive status, number of drugs, Charlson Comorbidity Index, ASA classification, type of surgery, use of cardiopulmonary bypass, general anesthesia yes/no, cut-to-suture-time, IMC/ICU stay, and preoperative creatinine. Significant p-values are marked in bold*.

One of the confounding variables we included in the adjusted regression analysis (Table 3) was the ASA physical status classification, which is widely used to assess pre-anesthesia medical comorbidities. The Barthel Index is commonly applied to assess the functional status of older patients. Thus, we additionally performed an adjusted regression analysis using the Barthel Index instead of the ASA classification. The ORs for both, ARS (OR = 1.37, 95% CI = 1.04–1.81) and ABS (OR = 2.44, 95% CI = 1.39–4.28), changed only slightly. This result confirms that neither physical status (ASA) nor functional status (Barthel Index) accounted for the increase of POD with anticholinergic medication.

## Discussion

Preoperative anticholinergic medication exposure was assessed by two different scales estimating the overall anticholinergic burden, the weighted ARS and the more recent ABS. The findings of this study suggest that anticholinergic medication usage might be an independent risk factor for POD in older population undergoing elective surgery. The reduction or omission of these drugs preoperatively might thus be beneficial for the prevention of POD.

Our results are in line with several previous studies, which reported an association between anticholinergic drug exposure measured with the ARS and the occurrence of delirium in older patients (38–41). A recently published systematic review by Egberts et al. showed that among the variety of existing anticholinergic drug scales, the Anticholinergic Risk Scale (ARS) was the only one found to be consistently associated with delirium (22). Also, our findings are in accordance with a published study by Mueller et al. who showed an independent association between preoperative anticholinergic burden and the occurrence of POD (prevalence 10%) in a sample of older cancer patients (mean age 71 years, *n* = 651) (23). Yet, in contrast to our study, this study used the Anticholinergic Drug Score (42) to determine the anticholinergic burden. Other studies did not find an association between anticholinergic burden and delirium (43, 44). Reasons for the inconsistent results of the studies are manifold. One reason could be that the measurement of the anticholinergic load has not yet been standardized (22). This leads to a large body of existing literature concerning DAPs and a lack of consensus on how to quantify the anticholinergic burden. In recent years, numerous different scales have been developed. However, they vary widely in their structure, focus, application, measurement of anticholinergic properties (serum vs. predefined) and association with outcomes (21). Therefore, comparability of individual study findings is limited, and an international consent on the most feasible instrument is strongly needed. The use of different tools to determine the anticholinergic burden is one explanation for the inconsistent findings of previous studies. Furthermore, there is a large heterogeneity in study populations comprising residents of nursing homes (38), Australian veterans (45), Taiwanese National Health Insurance database (46), palliative care inpatients (39) and acutely ill hospitalized patients (20, 40), making it even more difficult to compare results. Moorey et al. claimed that delirium is not associated with the anticholinergic burden in older patients on admission to an acute hospital (43). In contrast to our study, they used the Anticholinergic Cognitive Burden Scale (47) and the Anticholinergic Drug Score which were both not consistently associated with delirium (22). Pasina et al. also used the Anticholinergic Cognitive Burden Scale in their recently published study and did not find a clear association between anticholinergic burden and delirium in patients admitted to an acute geriatric ward (44). Also, in contrast to our findings, a recently published study by Heinrich et al. found no association between preoperative anticholinergic load and POD using the Anticholinergic Cognitive Burden Scale, the Anticholinergic Drug Score and the ARS (24). However, compared to our study, these patients were significantly younger (POD: 74 years, no POD: 71 years in median) and patients with more than mild cognitive impairment (Mini-Mental State Examination score ≤ 23 points) were excluded.

As mentioned above, to our best knowledge, this is the first study using the ABS to explore the effect of DAPs on delirium. In comparison to the ARS, ORs for occurrence of POD were clearly higher using the ABS than the ARS. Our results suggest a 2.7-fold risk of developing a POD for each drug included in the ABS. Therefore, our findings provide a strong argument for modifiable delirium risk assessment by ABS in older patients scheduled for surgery. The PAWEL study's intervention bundle AKTIVER (Alltags- und Kognitions-Training & Interdisziplinarität verbessert Ergebnis und mindert das Risiko [“everyday skills and cognition training and interdisciplinarity improves outcome and mitigates risk”]) has been shown to reduce delirium by 33% in patients undergoing elective orthopedic and abdominal surgery by daily application of individualized modules on activation, relaxation and diagnostic chaperonage during the hospital stay (37). In addition to those actions for delirium prevention, software programs, or an app could be implemented into the hospital clinical information system to raise awareness of detrimental drugs even before surgery of older patients to enable discontinuation or substitution prior to anesthesia to avoid postoperative delirium. In most cases, alternatives more appropriate for older people are available or medication is not crucial during the vulnerable perioperative period. However, further studies are required to validate ABS as a useful tool to reduce the risk of POD in older patients in addition to the AKTIVER bundle.

## Limitations and Strengths

This study has several limitations. First, our approach to measure anticholinergic drug exposure did not include the dosages of DAPs. It is quite conceivable that higher dosages of DAPs have a stronger negative impact on the development of POD. Second, the ARS was developed in 2008 and was not updated since then. This could lead to an underestimation of anticholinergic burden due to an abandonment of newer DAPs. Third, we collected our information from patients' medication lists and verbal information but had little information about adherence to prescriptions prior to hospital admission. Finally, we did not consider delirium severity and duration in this study. The strengths of our study are the prospective multicenter study-design, the large number of patients and the usage of two different tools to measure anticholinergic burden. In addition to the established ARS as a weighted score, we used ABS as a quick and simple instrument to quantify anticholinergic burden in delirium patients for the first time. A further strength is the relatively high number of strong confounders like cognition included in our multivariable regression analysis.

## Conclusion

This study of 899 older patients undergoing various elective surgical procedures shows that the preoperative anticholinergic burden, assessed by ARS or ABS, is an independent risk factor for POD in older patients. Delirium occurrence was more than 2.7 times higher if a patient took at least one of the 56 drugs listed in the ABS (16), even after controlling for the most known delirium risk factors. The POD rate increased from 21.7 to 42.3% in patients receiving one or two ABS drugs. Identifying DAPs prior to hospital admission might be an opportunity to terminate or substitute anticholinergic drugs preoperatively and prevent delirium after elective surgery.

## Data Availability Statement

The raw data supporting the conclusions of this article will be made available by the authors, without undue reservation.

## Ethics Statement

This study was approved by the Ethics Commission of the Faculty of Medicine of the Eberhard-Karls University and University Hospital Tübingen with number 233/2017BO1 on October 12, 2017 and by the Ethics Commission of the University of Potsdam with number 38/2017 on December 11, 2017. The study was registered on the German Clinical Trials Register (DRKS-ID: DRKS00012797) in July, 2017. The patients/participants provided their written informed consent to participate in this study.

## Author Contributions

MH, CT, and GE designed this secondary analysis of the PAWEL-Study, planned the data collection, performed the statistical analysis, and prepared and revised the manuscript. CB, CM, FK, EM, SW, LC, BF, MR, CA, and MD were involved in data collection and critically revised the manuscript for final approval of the version to be published. All authors contributed to the article and approved the submitted version.

## Funding

This study was funded by the Innovationsfonds (Fund of the Federal Joint Committee, Gemeinsamer Bundesausschuss, G-BA; AZ: VF1_2016-201), which had no role in the design of the study and had no role either during its execution, analyses of the data, or in the decision to submit any results. We acknowledge support by the Open Access Publication Fund of the University of Freiburg.

## Conflict of Interest

The authors declare that the research was conducted in the absence of any commercial or financial relationships that could be construed as a potential conflict of interest.

## Publisher's Note

All claims expressed in this article are solely those of the authors and do not necessarily represent those of their affiliated organizations, or those of the publisher, the editors and the reviewers. Any product that may be evaluated in this article, or claim that may be made by its manufacturer, is not guaranteed or endorsed by the publisher.
